# Efficacy of Management Efforts to Reduce Food-Related Dingo–Human Interactions and Conflict on K’gari (Fraser Island), Australia

**DOI:** 10.3390/ani13020204

**Published:** 2023-01-05

**Authors:** Linda Behrendorff, Rachel King, Benjamin L. Allen

**Affiliations:** 1 Queensland Government Department of Environment and Science, Queensland Parks and Wildlife Service, K’gari (Fraser Island), QLD 4581, Australia; 2 School of Mathematics, Physics and Computing, University of Southern Queensland, Toowoomba, QLD 4350, Australia; 3 Institute for Life Sciences and the Environment, University of Southern Queensland, Toowoomba, QLD 4350, Australia; 4 Centre for African Conservation Ecology, Nelson Mandela University, Port Elizabeth 6034, South Africa

**Keywords:** *Canis lupus* dingo, Fraser Island, habituation, risk, wild dog, supplementary feeding, anthropogenic impacts, food webs, canid

## Abstract

**Simple Summary:**

Improved understanding of the drivers of human–predator conflict may assist in reducing such conflicts. We collated available human–dingo interaction reports from K’gari (Fraser Island) since 1990, and provide an analysis of these interactions. We show where food or access to food has influenced dingo behaviour, and identify any changes in the rate of food-related interactions over time, thereby determining if management actions are achieving their intended goals or having unintended consequences. Our results support the view that food provisioning can influence the behaviour of canid predators in relatively intact ecosystems and that nonlethal management actions that seek to prevent access to anthropogenic food can produce measurable reductions in food-related interactions over time.

**Abstract:**

Humans and dingoes (*Canis familiaris* (dingo)) share the environment of K’gari, and conflict inevitably occurs between the two species, particularly over food. Dingo attacks on humans have occurred, and some have been serious and even fatal in outcome. Wildlife feeding may cause animals to develop unnatural and potentially dangerous behaviours towards conspecifics and humans on a relatively frequent basis. Food-based attraction has been implicated in the development of human-directed aggression in the dingo population of K’gari. Supplemental feeding, whether intentional or accidental, alters wildlife foraging behaviours and may have consequences at the population and ecosystem levels. Management strategies such as education programs, prohibition of inappropriate human behaviours (compliance) and fencing of garbage dumps have each been implemented to stop the intentional or inadvertent feeding of dingoes by people. However, there has been no formal assessment of the effectiveness of these interventions at reducing food-related dingo–human incidents over time. We collated and analysed 7791 unique reports of dingo–human interactions on K’gari between 1990 and 2020, inclusive of 1307 food-related reports, including the severity of these interactions. These data showed clear seasonal peaks in the percentage of food-related dingo–human interactions, corresponding with biologically significant breeding periods in autumn and weaning and dispersing in spring. Trends in serious food-related incidents remained stable overtime. Less serious food-related incidents declined, suggesting that management efforts were successful. However, these efforts appear to have reached the limits of their effectiveness. Further innovations are required to reduce serious incidents involving the relatively few dingoes and people still experiencing conflict, and thereby provide protection to both species on K’gari.

## 1. Introduction

Conflicts between humans and wild animals occur throughout the world and are defined as actions or reactions by one that have a negative effect on the other. For humans, human–wildlife conflict is often experienced as a loss of amenity, property, income and occasionally loss of life [1,2,3]. For wildlife, this conflict typically results in expulsion and exclusion from human-dominated areas, coupled with forms of control that often lead to individual death and population decline [4,5]. Managing and resolving these conflicts are important for the coexistence of both humans and wildlife and remain a key priority for many communities around the world [6,7].

The relationship between humans and dingoes (*Canis familiaris* (dingo)) in Australia is a classic example of human–wildlife conflict. After arriving in Australia about 250 years ago, Europeans quickly discovered dingoes’ ability to decimate introduced sheep and goat populations [8]. Disdain for dingoes grew with European settlement across the continent and continues today in all areas where livestock are grazed. Dingoes also impact people in urban areas [9] and have been involved in lethal attacks on humans [10,11]. In response, lethal control of dingoes in one form or another now occurs over approximately half of Australia and dingoes have been eradicated or excluded from approximately 15% of Australia [12]. In other areas, dingoes are highly valued as important species, both for their ecological roles and their aesthetic and cultural value. This includes K’gari (Fraser Island) [13,14], situated a short distance off the south-east coast of Queensland, where dingoes are protected as an iconic species and are culturally significant to the Butchulla [Badjala] people, who hold strong affiliations and connections to dingoes, known locally as wang’ari [15]. They are important to the island’s cultural, conservational and ecological values. However, this has not prevented people intentionally (e.g., throwing food to dingoes) and inadvertently (e.g., leaving food unattended) feeding dingoes and regularly interacting with them in ways harmful to both dingoes and humans [16,17,18,19]. These interactions culminated in the death of a young boy in 2001, and the deaths of many dingoes since this time as managers grapple with balancing human–dingo conflict on the island [20].

The ultimate causes or drivers of these conflicts are contested. Feeding or providing resource subsidies to predators alters their behaviour and ecology and is generally considered to be a bad thing [21,22], particularly for dingoes [18,19,23,24,25,26]. Schmidt and Timm [27] reviewed the causes of this type of conflict for domestic dogs (*Canis familiaris*), coyotes (*Canis latrans*), dingoes and wolves (*Canis lupus*) and concluded that important factors leading to these conflicts included (1) an attractive, resource-rich environment, (2) human acceptance or indifference to predator presence, (3) a lack of understanding of predator ecology and behaviour, (4) intentional feeding and (5) a reduction or cessation of predator management programs. In their review of the ‘attraction > habituation > interaction > aggression model’ and analyses of individual dingo management histories, Allen and colleagues [28] (p. 80) reported that the provision of food (either deliberate or inadvertent) is a major contributing factor that ultimately leads many dingoes towards involvement in dangerous dingo–human interactions, and “while habituation does not always lead to aggressive behaviour, dangerous behaviour is always associated with habituation”. Further describing this ‘pathway to intervention’, Behrendorff [29] concluded that “familiarisation or attraction leads to habituation or human-tolerance, and without intervention, human tolerance leads to increasingly negative and higher-risk interactions, ultimately requiring intervention or humane destruction to avert a serious incident or attack.” Behrendorff [29] further stated that conflicts do not arise randomly amongst the dingo population, but predictably by animals that have followed this pathway. Some dingoes move through these stages over a period of months, while others can progress through these stages in less than a week. However, “by intervening early to prevent familiarisation and human-tolerance, managers can prevent dingoes from developing high-risk behaviours, thereby protecting dingoes and people from otherwise inevitable harm”.

**Figure 1:**
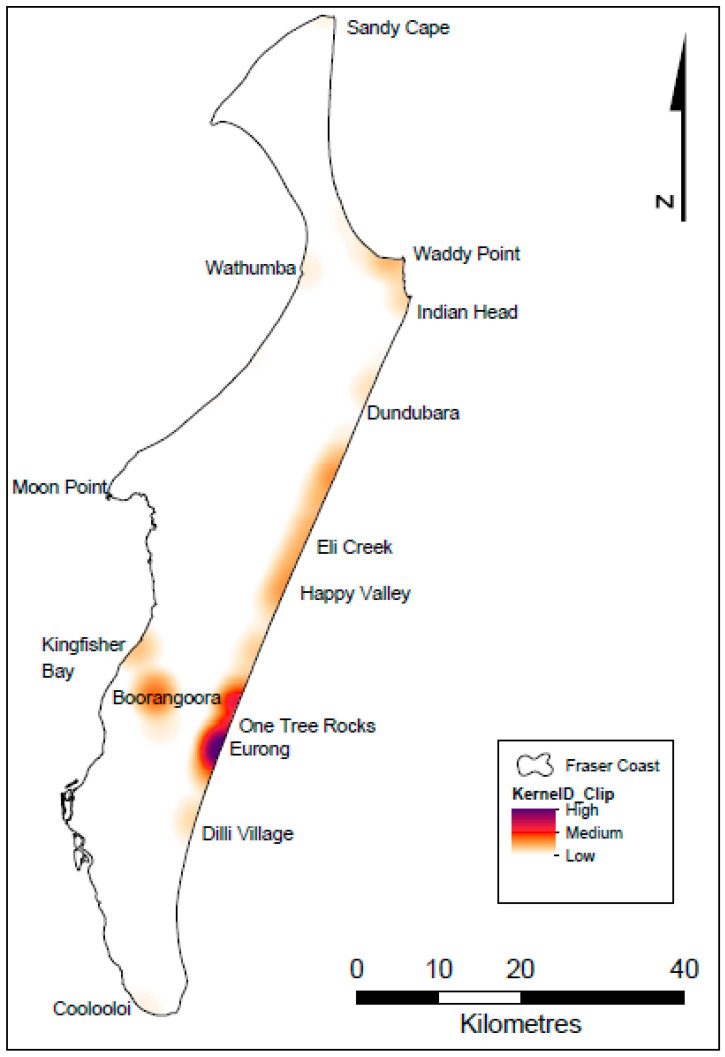
Geographic distribution and density of 7791 dingo–human incidents on K’gari (Fraser Island), 1990–2020. Map created in ArcGIS.

There is a general consensus in the literature discussing the ultimate drivers of dingo–human conflict on K’gari, which supports the food-driven model as the most parsimonious one to describe how humans create the conflict, but alternative views have been offered. For example, O’Neill and colleagues [30] proposed the “cull > social disruption > elevated breeding rates and dispersal > conspecific conflict and ecological decline > stress and aggression > human conflict > further culls” model to explain why dingoes interact negatively with humans, asserting that if managers simply stopped euthanising dangerous dingoes (which occurs at a rate of about 1.8 dingoes per year; [31], but see also Allen and colleagues [28]), then human–dingo conflicts would cease. Though a variety of complex and interacting factors undoubtedly contribute to the development of conflicts, in line with Corbett [32], Appleby and colleagues [17] also conclude that “habituation, and particularly feeding, may set the stage for incidents to occur” (see also Appleby [10]). Managers have, therefore, continued to actively implement actions that seek to reduce food availability and food-related interactions (Figure 1). However, whether or not these management actions have succeeded in reducing food-related interactions has not been thoroughly explored. Investigating “human–dingo behaviour and interactions, including causes and drivers of incidents” remains a key management recommendation, because such knowledge could then “be used to inform educational messages regarding how people should behave in relation to dingoes” (Allen and colleagues [28] (p. 113)).

Here, we explore temporal patterns in food-related dingo–human interactions on K’gari. The collation and curation of a comprehensive interaction dataset allows for the assessment of the food-related recorded interactions within the context of the actions that managers have undertaken to reduce these types of interactions. Our aim is to identify any changes in the rate of food-related interactions over time and determine if management actions are achieving their intended goals or having unintended consequences. Through this, we hope to better understand the drivers of human–predator conflict and assist with reducing these conflicts into the future.

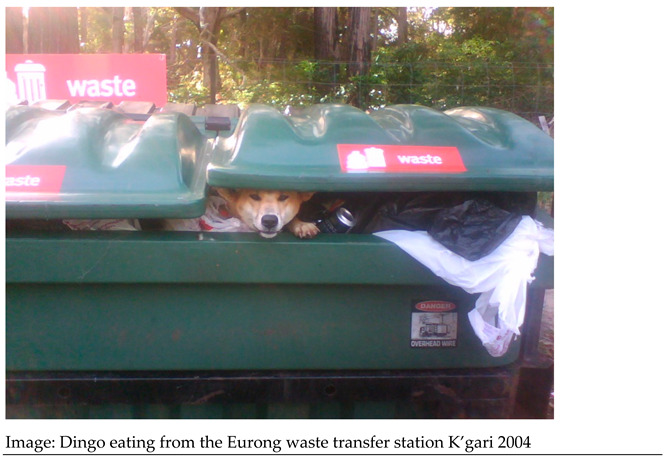



## 2. Materials and Methods

Study site

K’gari (Fraser Island) is situated approximately 1.5 km off the south-east coast of Queensland, Australia, within the Great Sandy National Park (Figure 1). The K’gari section of the park is co-managed by the Queensland Parks and Wildlife Service (QPWS) and the Butchulla Aboriginal Corporation (BAC). The 1640 km^2^ island supports a stable population of 100–200 dingoes [29]. About 500 residents also live within four major townships and the island attracts over 400,000 visitors per year [33]. A cumulative total of approximately 1% of the island is fenced off to restrict dingo access to a small number of townships, campgrounds, visitor eating areas and waste facility stations. Only ~5% of the island is accessible to the general public. Further details on the nature of the study site can be found elsewhere (e.g., [18,34,35]).

Interaction data

Reports describing interactions between dingoes and humans have been collected since 1990 and are now routinely collated into a database maintained by QPWS. Interaction reports are primarily used for management purposes to inform visitor risk assessment processes, but several studies have used these interaction reports for various research purposes in the recent past (e.g., [10,17,28,30]). Interaction reports are submitted to QPWS opportunistically by island visitors and residents, and are also actively solicited by QPWS rangers when talking with campers, fishers, tourists, QPWS staff and others. Curation of the interaction reports database is ongoing, and the database is regularly updated with new reports as they arise and whenever older hard-copy reports are found in archived boxes of historical material. As such, the database that we collated and curated for the present study represents the most comprehensive and accurate information available and will be slightly different from previous versions of the database used in prior studies. Inconsistent effort in collection of interaction reports, the knowledge that some interactions are unreported, and the imprecise quantification of visitor numbers means that attempts to calculate interaction rates over time can be unreliable [28]. As such, we cannot calculate interaction rates per time period, but instead focus on frequencies and proportions/percentages of interaction event types at several temporal scales (e.g., year, season and time of day).

Interactions are classified as Code A-, B-, C-, D- or E-type interactions [28], and a glossary of terms is used to allocate a code to the interaction behaviour displayed by dingoes. Code A and B interactions pertain to benign sightings of dingoes only (Code A interactions), or observations of dingoes behaving appropriately near people (Code B interactions). Code C interactions represent nuisance behaviour such as loitering around campgrounds, climbing on tables or vehicles, rummaging through containers or taking food items. Code D interactions represent more serious behaviours, including threatening behaviour such as growling or snarling at people; corralling people while eating, walking or fishing; or entering tents while people are inside. Code E interactions reflect the most serious types of interaction, and include biting people, chasing people or attacking people. Code A and B interactions are now recorded elsewhere within observation records and are widely unreported or underreported, so the current database comprises mostly Code C interactions, with smaller numbers of Code D and E interactions. 

We accessed available databases and obtained all available records of dingo–human interactions that occurred between 1990 and 2020 to create a more up-to-date database of interaction records. We cross-checked these with the database recently used by Allen and colleagues [28] and Appleby and colleagues [17] to ensure consistency and remove any duplicates. We then read through the entire database to correct any spelling mistakes and standardise formats for time, date and location. Most of these fields were complete for most reports, although a small number of reports had missing times or did not record the relevant Code. Where we felt confident at reliably doing so, we retrospectively assigned a Code to these reports based on the descriptive information provided in the report, cross-checking these few assigned codes amongst coauthors, the glossary of terms and QPWS colleagues. Where the exact time or hour of the interaction was not recorded, we also attempted to assign them to an hour based on the descriptive information in the report. For example, ‘early morning’ was assigned to 05:00 am, ‘mid-morning’ was assigned to 10:00 am, ‘dusk’ was assigned to 18:00 pm, ‘evening’ was assigned to 19:00 pm, and so on. Interaction reports that just recorded ‘daytime’ where assigned an hour of 12:00, which, given the large number of such records, produced the false impression that a great many incidents occurred at noon. Accordingly, we ignored values for this hour when assessing patterns in hourly trends (see below). We left all missing fields blank where we were not confident in assigning Codes or hours.

To extract reports that described food-related dingo–human interactions, we searched the entire revised database for the following keywords: access, ate, bag, bait, barb, BBQ, bin, box, bucket, camper, consume, container, cupboard, eat, esky, fed, feed, food, fridge, kitchen, lid, locker, open, packet, raid, rubbish, sealed, snatch, steal, stole, swallow, take, took. The identified reports were then read in detail to exclude any reports that were not truly food-related, such as those that reported an interaction such as ‘the dingo ran past the kitchen but did not steal any food’. We then assessed hourly, monthly, annual and seasonal trends in the proportion of food-related incidents, and how seasonal and annual trends differed for less serious (Code B and C) and more serious (Code D and E) interactions. Reports without an available or created code (*n* = 10) were omitted from interaction analyses.

## 3. Analysis

Logistic regression was used to consider whether the proportion of food- vs. non-food-related incidents was associated with the Year or the Season within which the incident occurred. Only the years from 2001 to 2020 inclusively were considered, given that the number of recorded incidents was much lower prior to 2000 when management and monitoring practices changed, and resources increased during 2001. For this analysis, the data were categorised as Breeding (March, April and May), Denning (June, July and August), Whelping (September, October and November) and Dispersal (December, January and February) seasons. The denning season was used as the reference season in all models. Three models where defined: the first considered all food-related incidents compared to all non-food-related incidents, the second considered only Code B- and C-type food and non-food incidents, and the third considered only Code D- and E-type food and non-food incidents. For all models, there were no significant interactions between Year and Season, and therefore only the results for the main effects of Year and Season are presented. Nonparametric Kendall’s tau-b correlation analysis was undertaken to further explore the direction and annual variability of trends. All analyses were conducted in R Studio [36] using R software [37]. 

## 4. Results

Our efforts to collect, collate and curate all available hard-copy and electronic interaction records produced a total of 7791 unique interaction reports for the period 1990 to 2020, from all across the island (Figure 1; Table 1). Of these records, 1307 were identified as being food-related (see Supplementary Material), leaving 6484 records that were not considered to be food-related (Table 1). Of the 1307 food-related records that we found, an hour of the day was available or created for 1083 of them. An interaction Code was not available or not created for only 10 of the 7791 records. The mean annual proportion of incidents that were food-related was 0.14, or in other words, 14% of interactions each year were food-related, on average. Of these, the mean annual proportion of benign Code B and C incidents that were food-related was 0.24, and the mean annual proportion of serious Code D and E incidents that were food-related was 0.03 (Table 1).

The logistic regression analysis (Table 2) showed that the proportion of all food-related interactions (compared to non-food-related incidents) significantly decreased from 2001 to 2020 (OR = 0.96, *p* < 0.001). There was also a significant increase in food-related interactions in the Breeding (OR = 1.29, *p* = 0.004), Whelping (OR = 1.21, *p* = 0.036) and Dispersal (OR = 1.30, *p* = 0.004) seasons compared to the Denning (reference level) season. This means that for each subsequent year from 2001 to 2020, the odds of an incident being food-related dropped by 0.04 (4%) on average, and a food-related incident was 1.29, 1.21 and 1.30 times more likely to have occurred in the breeding, whelping and dispersal seasons, respectively, than in the denning season, regardless of year.

When considering only Code B- and C-type interactions, the proportion of Code B- and C-type food related events again significantly decreased over the years (OR = 0.96, *p* < 0.001). The Breeding (OR = 1.28, *p* = 0.008) and Dispersal (OR = 1.32, *p* = 0.004) seasons also showed an increase in Code B- and C-type food related events compared to the Denning season; however, there was no significant change during the Whelping season (*p* > 0.05). When considering only Code D- and E-type interactions, the proportion of Code D- and E-type food-related events did not significantly change due to Year or Season (*p* > 0.05).

**Figure 2:**
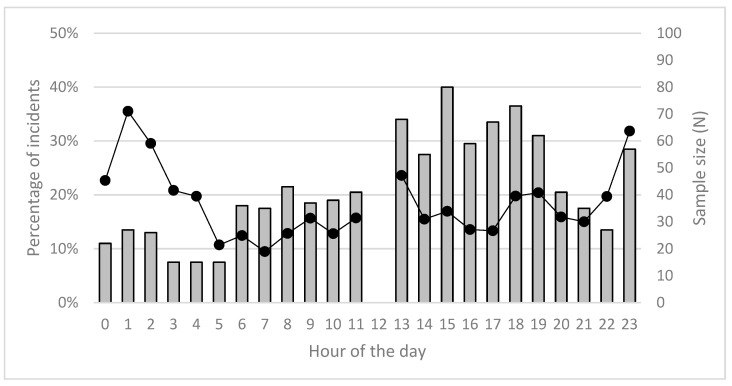
Hourly distribution (black marks and lines) and sample size (grey bars; N = 1083) of the percentage of food-related dingo–human interactions on K’gari, 1990–2020.

**Figure 3:**
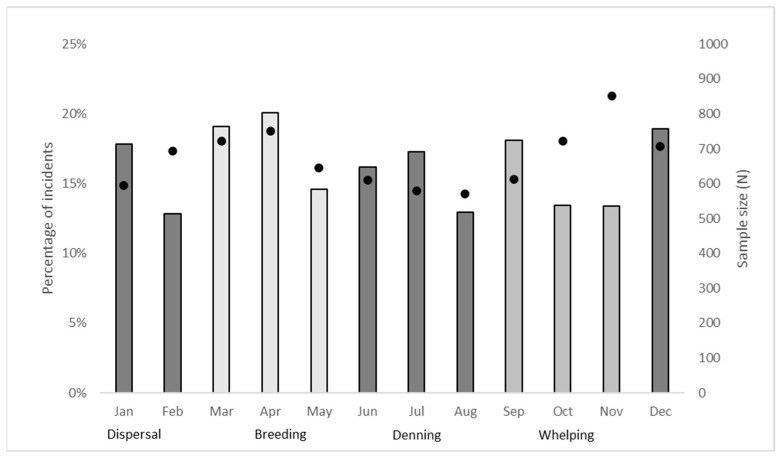
Monthly distribution and sample size (bars) (N = 1307) of the percentage (dots) of food-related dingo–human interactions on K’gari, 1990–2020.

There was no obvious pattern in food-related interactions over the 24 h cycle, although peaks seemed apparent at 13:00, 23:00 and 01:00 (Figure 2). Sample sizes were higher for afternoon and evening periods. In contrast, and in accordance with our earlier results (Table 2), there were two clear seasonal peaks in the percentage of food-related interactions in March–April and again in October–December, corresponding with the biologically meaningful periods of breeding in Autumn and whelping in Spring (Figure 3); see also Corbett [38].

**Figure 4:**
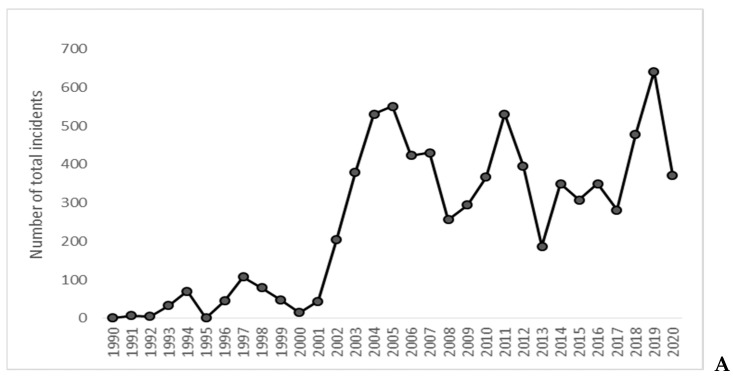

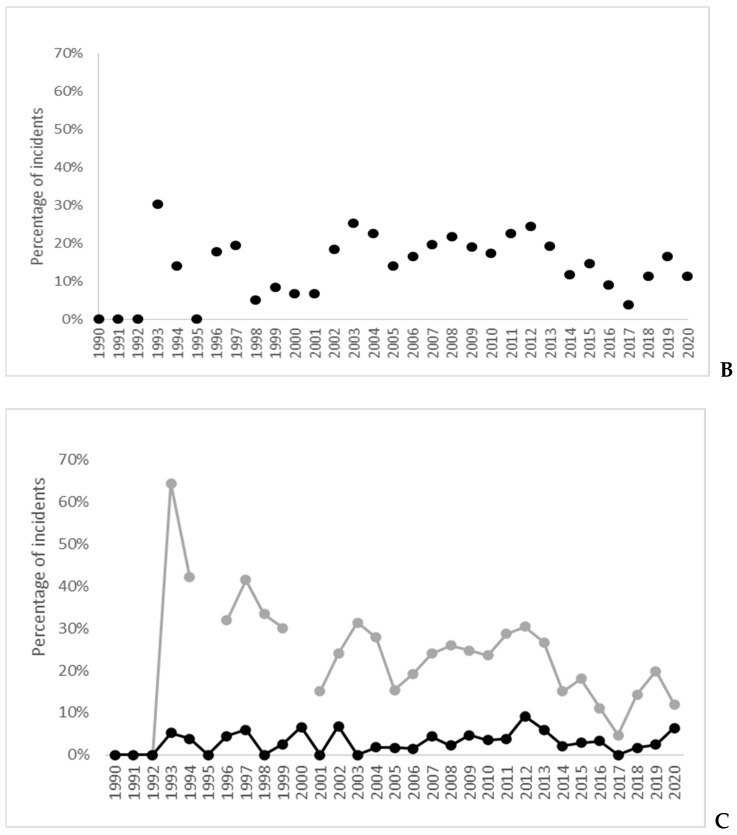
Annual trends in (**A**) the total number of reported dingo–human interactions on K’gari, 1990–2020, (**B**) the percentage of all food-related dingo–human interactions, and (**C**) the percentage of Code B and C interactions (grey dots and lines) and Code D and E interactions (black dots and lines) that were food-related.

Annual variation in the timing of these peaks was minimal (data not shown). Very few incidents were reported and/or recorded prior to 2001 (Figure 4A), making analyses of these data unreliable. Ignoring this period (or excluding data from 1990–2000), nonparametric Kendall’s tau-b correlation analyses suggested—but could not confirm—a monotonic decline in the percentage of food-related interactions over time (τ_b_ = 0.30, *p* = 0.064; Figure 4B). Separating Code B and C interactions from Code D and E interactions (Figure 4C) and rerunning this analysis produced a similar result for food-related Code B and C interactions (τ_b_ = −0.28, *p* = 0.086) but not food-related Code D and E interactions (τ_b_ = 0.15, *p* = 0.346). In other words, the percentage of Code D and E interactions that were food-related remained low and stable, and did not increase over time (Table 1 and Figure 4C), whereas the percentage of Code B and C interactions that were food-related was relatively high in the early 2000s (~20–30%), but declined to 12% by 2020. High annual variability in the proportion of food-related Code B and C interactions (Table 1; Figure 4C) contributed to our inability to confirm the decline in food-related interactions (Table 2) using this additional analytical approach. 

## 5. Discussion

Dingoes on K’gari are one of the most intensively managed populations of wildlife in Australia, and a great deal of effort is directed towards reducing their food-related interactions with people highlighted in Table 3. These efforts have largely been considered successful [29,33], but quantification of temporal trends in food-related interactions has not occurred until now. Our results reveal two seasonal peaks in food-related interactions (Figure 3, [12,38,39]), demonstrating a critical link between biologically meaningful periods of greater food demand and food-related dingo–human interactions. This was not apparent at finer temporal scales (Figure 2), but assessment of long-term trends reveals a decline in food-related interactions (Figure 4) associated with ongoing management efforts to reduce them.

Provision of food has long been thought of as an important driver of negative dingo–human interactions on K’gari [40], but confirmation of a biological link has been elusive. Allen and colleagues [28] showed that the frequencies of Code C, D and E interactions all peaked in March and April, and more focused analyses by Appleby and colleagues [17] confirmed this pattern for Code E incidents. This autumn period represents the time of year when peaks in raised-leg urination, defecation, howling, pairing-up, sperm and egg production, activity and movement also occur [38,39,41,42,43], and is a widely recognisable phenomenon of dingo biology. The October–November period is when the annual cohort of pups are weaned, emerge and begin to independently seek for food, disperse and integrate into a pack [44]. It might understandably be concluded that increased interactions at these times were biologically driven, but there has been some conjecture that these peaks (Figure 3) were a result of increased reporting or solicitation of interaction reports during holiday periods when more visitors are on the island—conjecture enabled by a lack of information on the amount of effort spent soliciting interaction reports at these times [28]. Had food not been a driver of interactions, however, then we would have expected to see no seasonal patterns in the proportion of food-related interactions, but this was not the case. Instead, we found clear seasonal peaks in the proportion of food-related dingo–human interactions in March–April and again in October–November (Figure 3) corresponding with the two most biologically significant periods of increased food demand, establishing a clear biological link between food and interactions.

Given the link between food and activity, we would anticipate that targeted management actions to reduce dingo access to human-sourced foods would result in a measurable reduction in food-related incidents. A variety of such actions have occurred since QPWS began specific dingo management on the island from 1990 (Table 3). These include closing unfenced rubbish dumps; provision of secure rubbish bins and food storage lockers at popular tourist locations and hiker camps; temporary campground closures during high-risk periods; implementation of strict compliance and enforcement activities or fines for feeding or disturbing dingoes; fencing townships and high-use visitor areas; prohibition of discarding bait and fishing waste on the beach; and ongoing collaborative education (see Madsen [45] and Bell [46]). These actions are ongoing and continue to be implemented across the island [47,48], which has resulted in a measurable decline in lower-risk Code B and C food-related interactions (Table 2 and Figure 4).

That trends in the proportion of food-related Code D and E interactions did not increase over time (Figure 4C) further suggests that management responses to persistent human–dingo conflict are not exacerbating the conflict. Some have proposed that restricting dingo access to anthropogenic food, restricting dingo movements by fencing-off townships and campgrounds and humanely destroying high-risk dingoes on occasion are all types of actions that are ultimately responsible for creating the conflict [30,49,50,51], but this view is inconsistent with the evidence available. These actions have continued and intensified over the last 30 years [28,29,33], yet the frequency of food-related incidents has declined (Table 2) and the frequency of serious Code D and E incidents have remained low and stable over time (Figure 4). Had intensive dingo management been driving serious interactions, then we would have expected an increase in serious interactions.

Though we are confident in our results and conclusions, our study was limited by the unavailability of precise visitor numbers to the island. This issue has hampered previous evaluations of management effectiveness [28], but does not yet appear to have been satisfactorily resolved. We were also limited to interaction records that had been opportunistically collected, and potentially influenced by reporting bias. Opportunistic sampling is largely unavoidable given the hundreds of thousands of visitors to the island each year, which cannot all be interviewed by QPWS staff. We acknowledge that this sampling strategy creates the potential for bias, but we do not expect it to occur systematically, given that interaction data were collected by multiple people over such a long time period. Regardless, our analysis of ‘trends in proportions’ instead of ‘rates of interactions’ effectively circumvented any possible confounding due to bias (if, indeed, there was any). Future studies seeking to address this issue might attempt to undertake a more complete survey of island visitors. Precise visitor numbers and complete sampling may greatly improve further assessment of interaction records and management effects on incident rates.

## 6. Conclusions

That the frequency of Code D and E interactions has not changed over time has led some to conclude that dingo–human interactions are not linked to food provisioning or that the aforementioned management actions have been ineffective at reducing serious incidents, even if they did not increase them [17,30]. While this latter point may be true, we instead speculate that it is not a failure of management actions to have an effect (when it has indeed had an effect; Table 2 and Figure 4), but rather that these successful management actions have instead reached the limit of their effectiveness. In other words, we consider it likely that all the management actions being implemented—as effective and necessary as they may be—are insufficient to dissuade the remaining small number of people from continuing to provide food to dingoes (e.g., [52]). Without further management innovation, we expect that current trends in the number of serious interactions (Figure 4; [17]) and number of dingo destructions (1–3 per year; [29,31]) will continue to trickle along as they have for the last decade or more. We agree with earlier conclusions ([28] pg. 80) that “deliberate or inadvertent feeding of dingoes by humans greatly increases the likelihood that certain individual dingoes, in certain situations, will attack people” and management efforts “should continue to focus on avoiding feeding of dingoes.” We further support management efforts to trial and develop new ways to address the last few people and dingoes that continue to cause conflict, such as aversive geofencing technologies and increased education and compliance activities [31].

Our results reveal important trends in food-related dingo–human interactions on K’gari and have important implications for managers. Provision of anthropogenic food subsidies has long been considered a key driver of dingo–human conflict, but some have speculated that the ultimate causes of this conflict are not food-related (e.g., [30]). Our findings do not support this view, but instead show a clear link between management activities that reduce the availability of anthropogenic food and temporal declines in the proportion of food-related interactions. Had food provisioning not been a causal factor for dingo–human interactions, we would not have seen such a response. Management actions to reduce food-related interactions have succeeded in reducing low-risk interactions, but further reductions in high-risk food-related interactions are unlikely to occur without further innovation targeted at the relatively few people and dingoes involved in ongoing conflict. In broader terms, our results support the view that food provisioning can influence the behaviour of canid predators in relatively intact ecosystems. Our results also support the view that nonlethal management actions that seek to prevent access to anthropogenic food can produce measurable reductions in food-related interactions over time. Continued efforts to do so may reduce human–dingo conflict and help maintain the pristine environment that the World Heritage-listed K’gari is renowned for—one where dingoes and visitors can both enjoy their experience in safety.

## Figures and Tables

**Table 1:** Sample sizes (N) and summary information for Figure 4.

	N Total Interactions	Proportion of Food-Related Interactions	Proportion of Code B and C Interactions	Proportion of Code D and E Interactions
N years with data	31	31	27	31
N incident records	7791	1307	5888	1893
Mean	251.32	0.14	0.24	0.03
Standard error	35.41	0.01	0.02	0.00
Median	282	0.15	0.24	0.03
Standard deviation	197.18	0.08	0.13	0.03
Range	640	0.30	0.64	0.09
Minimum	1	0	0	0
Maximum	641	0.30	0.64	0.09

**Table 2:** Regression analysis results considering the influence of Year and Season on the proportion of food interactions when considering all interactions, Code B- and C-type interactions, and Code D- and E-type interactions.

Incidents	Covariate	Estimate	Std Error	Z	Pr (>|z|)	Odds Ratio (95% CI)
All	Year	−0.04	0.01	−6.94	<0.001	0.96 (0.95, 0.97)
	Season (Breeding)	0.26	0.09	2.91	0.004	1.29 (1.09, 1.54)
	Season (Whelping)	0.19	0.09	2.10	0.036	1.21 (1.01, 1.45)
	Season (Dispersal)	0.26	0.09	2.87	0.004	1.30 (1.09, 1.56)
Code B and C	Year	−0.04	0.01	−7.15	<0.001	0.96 (0.95, 0.97)
	Season (Breeding)	0.25	0.09	2.65	0.008	1.28 (1.07, 1.54)
	Season (Whelping)	0.15	0.10	1.54	0.123	-
	Season (Dispersal)	0.28	0.10	2.87	0.004	1.32 (1.09, 1.60)
Code D and E	Year	0.01	0.03	0.37	0.708	-
	Season (Breeding)	−0.09	0.36	−0.26	0.793	-
	Season (Whelping)	−0.58	0.46	−0.13	0.201	-
	Season (Dispersal)	0.03	0.36	0.07	0.943	-

**Table 3:** Timeline of important management actions implemented to reduce food-related dingo-human interactions on K’gari, 1990–2021.

Year	Management Action
1990	QPWS become responsible for dingo management on Fraser Island
1994	Closure of the dump at Waddy Point
2000	‘Dingo smart’ education campaign initiated
2001	Campground facilities upgraded at Coolooloi, Central Station, Ungowa
	Fencing began on QPWS residences at Eurong, Ungowa, Waddy Point, Central Station
	Dingo-specific fines implemented for feeding and disturbing dingoes (On-the-spot $225, court imposed $3000)
	Dingo ranger program initiated
	Quarterly risk assessment reporting commenced
2002	Fencing completed at Waddy Point day-use area
	Campground facilities upgraded at Central Station, Lake McKenzie, Dundubara, Waddy Point, Ungowa, Wathumba, Lake Allom and Lake Wabby
	‘Be dingo-safe!’ education campaign initiated
	Instant dismissal policy implemented for resort staff caught feeding dingoes
	Permit conditions amended requiring all CTOs to lock food containers
	Restrictions implemented for fish cleaning and offal disposal
	Stainless steel lids installed on all public-use gas BBQ plates
2003	Fencing completed at Central Station, Dilli Village, Waddy Point, Garawongera
	Campground facilities upgraded at Lake Bowarrady, Moon Point, North Wathumba Spit
2004	Fencing completed at Kingfisher Bay and Dundubara
2005	Beach bins removed, replaced with fenced rubbish compounds
2006	Food storage facilities installed at all hiker camps
2008	Fencing completed at Happy Valley and waste station, Eurong Resort and Residential Valley
	Dingo-specific fines increased (On-the-spot $300, court imposed $4000)
2009	Fencing completed at Eurong waste station
	Wildlife photographer conviction for long term dingo feeding
2012	Fencing completed at K’gari camp
2014	Fencing completed at Cathedral Beach
2015	Commercial Tour Operators begin using temporary electric fencing around camps
2019	Dingo-specific fines increased (On-the-spot $2135, court imposed $10,676)
2020	Beachside campground fencing completed at Cornwalls, Wongai, Eli Creek and One Tree Rocks
2021	Dingo-specific fines increased (On-the-spot $2205, court imposed $16,138)
	Funding announced for fencing at Orchid Beach completed 2022
	Funding announced for fencing at Wathumba Campground completed 2021

## Data Availability

All data discussed in the paper can be located within the paper.

## Supplementary Materials

The following supporting information can be downloaded at: https://www.mdpi.com/article/10.3390/ani13020204/s1, Excel datasheet title. Supplementary Material_S1_Interaction Data.
